# The Inhibition of α-Glucosidase, α-Amylase and Protein Glycation by Phenolic Extracts of *Cotoneaster bullatus*, *Cotoneaster zabelii*, and *Cotoneaster integerrimus* Leaves and Fruits: Focus on Anti-Hyperglycemic Activity and Kinetic Parameters

**DOI:** 10.3390/molecules27207081

**Published:** 2022-10-20

**Authors:** Agnieszka Kicel, Anna Magiera, Marta Skrzywanek, Mariola Malczuk, Monika Anna Olszewska

**Affiliations:** Department of Pharmacognosy, Faculty of Pharmacy, Medical University of Lodz, 1 Muszynskiego St., 90-151 Lodz, Poland

**Keywords:** *Cotoneaster*, α-glucosidase, α-amylase, kinetic parameters, protein glycation, polyphenols

## Abstract

*Cotoneaster* species have gained significant importance in traditional Asian medicine for their ability to prevent and treat hyperglycemia and diabetes. Therefore, in this study, some aspects of the beneficial health effects of hydromethanolic extracts of *C. bullatus*, *C. zabelii,* and *C. integerrimus* leaves and fruits were evaluated, including their influence on α-glucosidase, α-amylase, and nonenzymatic protein glycation. The activity was investigated in relation to the polyphenolic profile of the extracts determined by UV-spectrophotometric and HPLC-PDA-fingerprint methods. It was revealed that all leaf and fruit extracts are a promising source of biological components (caffeic acid pseudodepsides, proanthocyanidins, and flavonols), and the leaf extracts of *C. bullatus* and *C. zabelii* contain the highest levels of polyphenols (316.3 and 337.6 mg/g in total, respectively). The leaf extracts were also the most effective inhibitors of digestive enzymes and nonenzymatic protein glycation. IC_50_ values of 8.6, 41.8, and 32.6 µg/mL were obtained for the most active leaf extract of *C. bullatus* (MBL) in the α-glucosidase, α-amylase, and glycation inhibition tests, respectively. In the kinetic study, MBL was displayed as a mixed-type inhibitor of both enzymes. The correlations between the polyphenol profiles and activity parameters (|*r*| > 0.72, *p* < 0.05) indicate a significant contribution of proanthocyanidins to the tested activity. These results support the traditional use of *Cotoneaster* leaves and fruits in diabetes and suggest their hydrophilic extracts be promising in functional applications.

## 1. Introduction

Diabetes mellitus is a civilization disease defined as a condition of chronically elevated blood glucose levels (hyperglycemia) with impaired carbohydrate, fat, and protein metabolism, resulting primarily from a deficiency of or the reduced effectiveness of endogenous insulin [1]. In diabetic patients, uncontrolled postprandial hyperglycemia leads to a progression of microvascular (nephropathy, neuropathy, retinopathy) and macrovascular (atherosclerosis, ischemic heart disease) complications. A crucial role in the pathogenesis of these disorders is played by the formation of advanced glycation end products (AGEs) in a nonenzymatic reaction between the carbonyl group of reducing sugars and the free amino groups of proteins [2]. Scientific evidence suggests that AGEs trigger intracellular signaling leading to the production of reactive oxygen species (ROS) and various cytokines/chemokines. The overproduction of these pro-oxidant and pro-inflammatory factors contributes to the development of chronic oxidative stress and inflammation, which leads to the degradation of functional biomolecules, resulting in irreversible damage and dysfunction of numerous body organs [3]. One therapeutic approach in treating diabetes is the reduction of postprandial hyperglycemia by delaying glucose absorption through inhibiting carbohydrate-hydrolyzing enzymes such as α-glucosidase and α-amylase in the gastrointestinal tract [4]. Another way of treating diabetes, independent of glycemic control, is to prevent the formation of AGE products, which, by reducing pathological protein changes, leads to the prevention of diabetic complications. The proven effectiveness of AGE inhibitors is a result of their antioxidant properties and ability to chelate metals and interact with proteins and/or block the receptors of advanced glycation end products [5,6].

Traditionally, plant extracts are used to manage diabetes [7,8]. Antidiabetic agents derived from natural resources can be an alternative to synthetic hypoglycemic drugs, which are not always satisfactory and have side effects [9]. The antidiabetic activity of plant-derived extracts is usually attributed to nonnutrient phytochemicals, including phenolic compounds [7,10,11,12]. These specialized plant metabolites, depending on their structure, have the ability to inhibit carbohydrate-hydrolyzing digestive enzymes, improve glucose transport, stimulate insulin secretion from pancreatic β-cells, reduce insulin resistance, increase insulin sensitivity, reduce the production of AGEs, and enhance antioxidant defense [11,12,13].

Promising candidates capable of attenuating diabetes might be phytocompounds present in different parts of *Cotoneaster* species (Rosaceae) that are often linked to preventing civilization diseases [14]. Indeed, the herbal remedies provided by the genus *Cotoneaster* are popular in Asian traditional medicine, primarily in treating diabetes mellitus and its cardiovascular complications. The therapeutic use of *Cotoneaster* herbals is associated with their blood sugar-regulating capacity and hypotensive, diuretic, and anti-spasmodic properties [14,15,16,17]. However, their potential bioactivity mechanisms related to the treatment of diabetes are still insufficiently recognized and limited in literature to only a few studies [17,18,19]. According to the previous research, polyphenol-rich ethyl acetate, methanolic, and aqueous extracts of *C. nummularia* leafy twigs were effective inhibitors of α-glucosidase and α-amylase, i.e., the major enzymes involved in the digestion of carbohydrates [17]. In a comparative in vitro study of *C. integerrimus* fruits and leafy twigs, the tested methanolic extract of twigs reduced the activity of α-glucosidase more effectively than acarbose, a standard antidiabetic medication [18]. The supplementation of mucilage fraction of *C. horizontalis* leafy twigs, rich in monosaccharides such as glucose, xylose, arabinose, and rhamnose, at a 250 mg/kg dose once a day for 28 days also significantly regulates the levels of blood glucose and glycated hemoglobin HbA1c in streptozotocin-induced diabetic rats [19]. Several reports indicate the potential of *Cotoneaster* plants as effective oxidative stress reducers in chemical and biological models in vitro [20,21]. Significant antioxidant activity as a crucial health-promoting factor was previously indicated for the leaves and fruits of *C. bullatus*, *C. zabelii*, and *C. integerrimus* [20,21,22]. Several studies documented that oxidative stress plays a pivotal role in the pathogenesis of diabetes and its complications, especially within the cardiovascular system, categorized as macro- and microvascular lesions [23]. The generated oxidants are involved in developing vascular complications through endothelial dysfunction and inflammation [24]. In this context, the previously preselected leaves and fruits of *C. bullatus*, *C. zabelii*, and *C. integerrimus* may have a beneficial effect on reducing oxidative stress in diabetic patients and significantly reducing the disease severity and complications. However, due to scarce previous research, a deeper study of the inhibitory effects of these herbals on glycolytic enzymes, kinetic inhibition parameters, and antiglycation properties is required to evaluate their value as antidiabetic agents and verify their possible mechanisms of action.

Therefore, the aim of the present study was to analyze for the first time some aspects of the anti-hyperglycemic activity of the *C. bullatus*, *C. zabelii*, and *C. integerrimus* leaves and fruits, which may explain their traditional use in the treatment of diabetes mellitus. To select the leaves and fruits with optimal quality in terms of phenolic composition and activity, the phytochemical profiling of their methanol–water (7:3, *v/v*) extracts was carried out using HPLC-PDA-fingerprint and UV-spectrophotometric methods. Next, the leaf and fruit extracts were subjected to evaluation of their inhibitory effects on α-glucosidase and α-amylase. For the most active extract, an enzyme kinetic study using Michaelis–Menten and Lineweaver–Burk plots was investigated to understand the possible mechanisms of the *Cotoneaster* polyphenols’ impacts on the digestive enzymes studied. In addition, the inhibitory effects of the extracts on the formation of advanced glycation end products AGEs were investigated. Finally, correlation studies were used at all study stages to evaluate the contribution of the extract polyphenols to the observed activity effects.

## 2. Results

### 2.1. Polyphenolic Profiling of Fruit and Leaf Extracts

**Figure 1:**
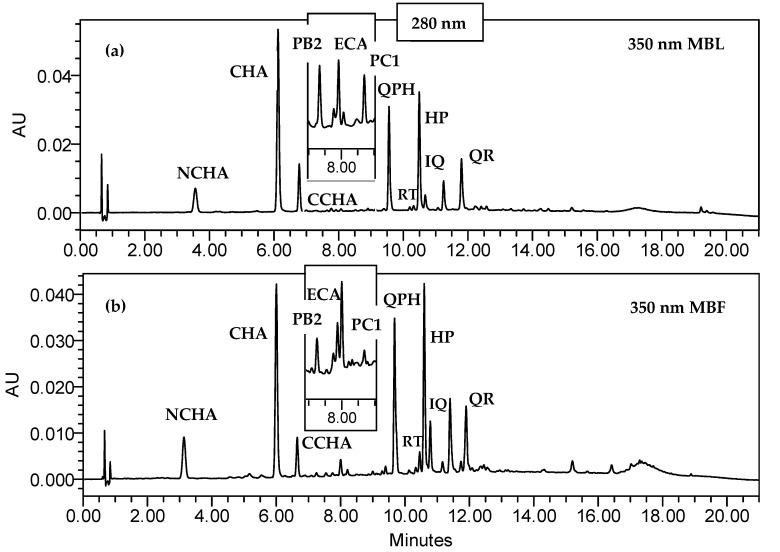
The representative HPLC-UV chromatograms of the methanol–water (7:3, *v*/*v*) extracts from the *C. bullatus* leaves MBL (**a**) and fruits MBF (**b**); the extract concentrations: 2.20 and 13.29 mg/mL for MBL and MBF extracts, respectively; for details of compound codes, see Table 1.

The qualitative LC-MS analysis showed that the dried methanol-water (7:3, *v/v*) extracts presented polyphenolic profiles identical to those observed for the leaves and fruits of *C. bullatus*, *C. zabelii*, and *C. integerrimus* in our previous phytochemical studies [20,21,22]. The data confirmed the tendency of the leaves and fruits to accumulate a wide range of active metabolites, including caffeic acid pseudodepsides and flavan-3-ols with (−)-epicatechin and its di-, tri- and tetramers, as well as mono- and diglycosides of flavonols (kaempferol and quercetin). The leaf and fruit extracts of each *Cotoneaster* species showed similar qualitative composition, while the observed differences were related to the proportions of individual components. The above observations were confirmed by quantitative spectrophotometric and HPLC-PDA analysis. As shown in Figure 1 and Table 1, the hydrophilic extracts of the leaves were significantly richer in polyphenols (TPC = 200.3–337.6 mg/g dw; TPH = 100.4–134.9 mg/g) compared with the fruits of the corresponding species (TPC = 62.1–81.3 mg/g; TPH = 12.2–22.3 mg/g).

The leaf extracts of *C. bullatus* and *C zabelii,* designated as the richest sources of polyphenols, were characterized by a predominance of oligo- and polymeric flavan-3-ols and caffeic acid pseudodepsides (UV-Vis and HPLC results). Procyanidin B2 and chlorogenic acid were the primary constituents of the *C. bullatus* leaf extract, while caffeoylmalic acid dominated in the *C. zabelii* leaves. In the case of *C. integerrimus* leaf extract, in addition to proanthocyanidins presenting in only in polymeric forms (UV-Vis results), significant proportions of flavonoids and caffeic acid derivatives were recorded. Among the individual polyphenols, the highest contents of chlorogenic acid and hyperoside were noted.

Compared with the leaf extracts, all fruit extracts of related species contained at least three times lower levels of polyphenols, with a predominance of polymeric proanthocyanidins in their composition (UV-Vis results). Based on a more detailed HPLC-PDA analysis, mono- and oligomeric flavan-3-ols (TLPA), caffeic acid derivatives (TCA), and flavonoids (TFL) were detected in the *C. bullatus* fruit extract at similar total levels. Among the individual polyphenols, the predominance of chlorogenic acid was noted. The fruit extracts of *C. zabelii* and *C.* integerrimus were distinguished by a high level of oligomeric flavan-3-ols, among which procyanidins C1 and B2 or (−)-epicatechin alternately dominated.

### 2.2. Effect of Leaf and Fruit Extracts on the α-Glucosidase and α-Amylase Inhibition

All *Cotoneaster* leaf and fruit extracts demonstrated a significant and concentration-dependent ability to inhibit in vitro yeast α-glucosidase and pancreatic α-amylase (Table 2). In the α-glucosidase inhibition assay with *p*-nitrophenyl-α-D-glucopyranoside (PNPG) as substrate, the leaf and fruit extracts showed 2–20 times higher activity than the anti-diabetic drug acarbose (IC_50_ = 169.5 µg/mL). Among them, the leaf extracts of *C. bullatus* and *C. zabelii* were the most effective (IC_50_ = 8.6 and 9.5 µg/mL, respectively) and their activity was 18–20 times higher than that of acarbose. In the group of standard polyphenols, the most effective α-glucosidase inhibitor was caffeoylmaic acid (IC_50_ = 43.7 µg/mL). In the α-amylase inhibition assay in the presence of potato starch, the studied leaf and fruit extracts showed variable effects, but the highest effectiveness was also noted for the leaf extracts of *C. bullatus* and *C. zabelii* (IC_50_ = 41.8 and 33.0 µg/mL, respectively). In contrast, all model polyphenols proved to be relatively weak inhibitors of α-amylase.

### 2.3. Mode of α-Glucosidase and α-Amylase Inhibition by Leaf Extract of C. bullatus

The type of enzymatic inhibition, i.e., competitive, noncompetitive, or mixed, was studied for the model extract of *C. bullatus* leaves using Lineweaver–Burk and Michaelis–Menten plots of enzyme kinetics [25,26].

**Figure 2:**
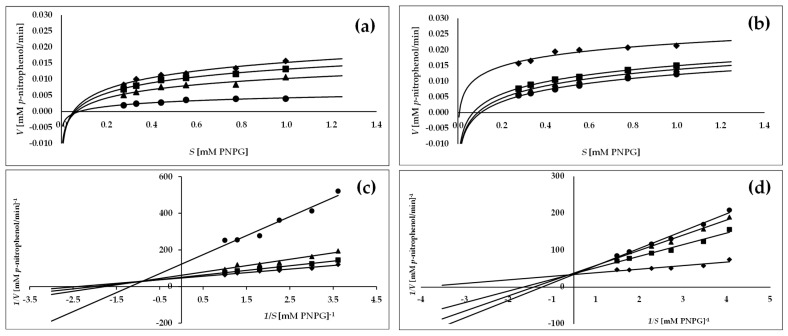
Michaelis–Menten plots (**a**,**b**) and Lineweaver–Burk plots (**c**,**d**) of methanol–water (7:3, *v/v*) extract of *C. bullatus* leaves MBL (**a**,**c**) and acarbose (**b**,**d**) for inhibition of α-glucosidase; the MBL concentrations: ♦—control (no inhibitor), ■—5.0 µg/mL, ▲—6.0 µg/mL, •—9.0 µg/mL; the acarbose concentrations: ♦—control (no inhibitor), ■—110 µg/mL, ▲—160 µg/mL, •—215 µg/mL.

**Figure 3:**
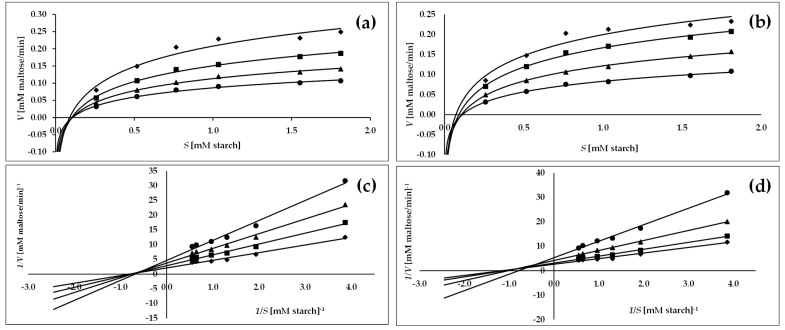
Michaelis–Menten plots (**a**,**b**) and Lineweaver–Burk plots (**c**,**d**) of methanol–water (7:3, *v/v*) extract of *C. bullatus* leaves MBL (**a**,**c**) and acarbose (**b**,**d**) for inhibition of α-amylase; the MBL concentrations: ♦—control (no inhibitor), ■—20.0 µg/mL, ▲—40.0 µg/mL, •—10.0 µg/mL and acarbose (**b**,**d**) with concentrations: ♦—control (no inhibitor), ■—3.0 µg/mL, ▲—8.0 µg/mL, •—10.0 µg/mL).

As shown in Figure 2c and Figure 3c, all activity lines in the Lineweaver–Burk plots intersected in the second quadrant, indicating changes in 1/*Vmax* (reaction rate) and 1/*S* (substrate concentration) with increasing concentration of *C. bullatus* leaf extract as the reaction inhibitor. It suggests a mixed inhibition type of both α-glucosidase and α-amylase [26]. However, considering the location of the intersection of the activity lines, which is closer to the X axis in the Lineweaver–Burk plots (Figure 2c and Figure 3c), it may be a mixed type of inhibition but with a tendency toward a noncompetitive type [26]. The observed mixed type of inhibition was also confirmed by the kinetic parameters reported in Table 3. An increase in the inhibitor concentration resulted in a significant decrease (*p* < 0.05) in *Vmax* (maximum velocity) with a simultaneous increase in *Km* (Michaelis constant), which is typical for the mixed type of inhibition [25,26]. In addition, in the case of α-amylase, both the extract and the reference acarbose revealed the same mixed type of inhibition (Figure 3b,d; Table 3).

### 2.4. Effect of Leaf and Fruit Extracts on NonEnzymatic Protein Glycation

All *Cotoneaster* leaf and fruit extracts exhibited a significant and concentration-dependent protective effect against nonenzymatic protein glycation in the bovine serum albumin (BSA)–fructose model in vitro (Table 2). The leaf extracts of *C. bullatus*, *C. zabelii*, and *C. integerrimus* were the most effective inhibitors of protein glycation (IC_50_ = 32.6–36.5 µg/mL), with capacity twice as high as that observed for aminoguanidine (IC_50_ = 71.1 µg/mL), the synthetic drug used clinically to treat diabetic complications and known to prevent the formation of AGEs. Apart from the extracts, the standard polyphenols, especially procyanidin B2 and (−)-epicatechin, showed high anti-AGE activity, significantly exceeding the effectiveness of aminoguanidine.

## 3. Discussion

Despite the increasing advances in modern medicine, plant-based remedies are still required to prevent and treat many chronic diseases, including diabetes and its cardiovascular complications. Postprandial hyperglycemia is an important initial characteristic of diabetes mellitus; therefore, inhibiting digestive enzymes such as α-glucosidase and α-amylase is one of the most effective ways to alleviate hyperglycemic conditions. The α-amylase catalyzes the hydrolysis of the dietary starch into maltose; this disaccharide is digested further by α-glucosidase, a specific membrane-bound enzyme in the small intestine. In addition, chronic hyperglycemia, contributing to AGE formation, plays a significant role in developing long-term diabetic complications such as diabetic retinopathy, nephropathy, cataracts, and atherosclerosis [6]. For this reason, natural inhibitors of α-glucosidase, α-amylase, and AGE products derived from plant extracts appear as promising therapeutic options to supplement or even substitute existing synthetic drugs.

In the present study, all investigated *C. bullatus*, *C. zabelii*, and *C. integerrimus* extracts were active against α-glucosidase and α-amylase. Furthermore, in all tests, the strongest inhibitory potential compared with the synthetic standard of acarbose was demonstrated for the leaf extracts of *C. bullatus* and *C. zabelii* (Table 2). Acarbose is one of the few commercially available inhibitors of carbohydrate-digesting enzymes, but it is also responsible for adverse gastrointestinal side effects. For this reason, some traditional and antidiabetic plants have already been studied to search for alternative natural drugs with fewer side effects and higher cost-effectiveness [4,7,10,27]. However, the literature review indicated that only a few available plant materials might achieve activity similar to those of *Cotoneaster* species analyzed in this study. For example, out of the 24 leaves from the Apocynaceae, Clusiaceae, Euphorbiaceae, and Rubiaceae families, only seven derived from *Willughbeia tenuiflora*, *Garcinia daedalanthera*, *G. kydia*, *Antidesma bunius*, *A. montanum*, *A. neurophalocarpum*, *A. malloticarpa*, and *Amensaracarpus pubescens* were able to inhibit α-glucosidase with IC_50_ in the range comparable to those obtained for *C. bullatus* and *C zabelii* leaves (IC_50_ = 8.6 and 9.5 µg/mL, respectively) [28].

As presented in Table 1 and Table 2, the antidiabetic activity of all *Cotoneaster* extracts strongly depended on the polyphenolic content, especially the levels of proanthocyanidins. Their close connection to the extract activity was confirmed by the statistically significant linear correlations between the levels of oligo- and polymeric flavan-3-ols and activity parameters; |*r*| > 0.89 and > 0.72, *p* < 0.05 for α-glucosidase and α-amylase, respectively (Table 4). According to the quantitative results (Table 1), the polyphenol contents in the most active leaf extracts of *C. bullatus* and *C. zabelii* reached 316.3 and 337.6 mg GAE/g, respectively, and about 45% of those values can be attributed to polymeric proanthocyanidins, which illustrates their dominant role in the extracts. This close correlation between procyanidins and activity parameters was also supported by the results of α-glucosidase inhibition for (−)-epicatechin and procyanidin B2 used as natural standards (Table 2). In comparison with the impact of flavan-3-ol derivatives on the extract activity, the effects of other extract components, such as caffeic acid pseudodepsides and flavonoids, were weaker (|*r*| < 0.38; Table 4). Nevertheless, the results in the α-glucosidase test (Table 2) for caffeoylmalic acid and quercetin mono- and diglycosides indicated their significant contribution to the capacity of the studied extracts, probably through additive and synergistic effects.

The protective effect of proanthocyanidins against diabetes was previously demonstrated in vitro and in vivo in animal models [29]. Furthermore, a recent study suggested that procyanidin-rich plant extracts inhibit digestive enzymes by forming enzyme-procyanidin complexes, which prevent the enzyme from interacting with standard polysaccharides like starch [30]. Most important, proanthocyanidins do not require gastrointestinal absorption to act in vivo as α-glucosidase inhibitors, since it is a membrane-bound enzyme present in the small intestinal epithelium. Hence, procyanidins, even with a high degree of polymerization, can exert local effects in the gastrointestinal tract as effective enzyme inhibitors [31].

Because of the various possible interactions between an inhibitor and enzyme, the present study also required an inhibition kinetics analysis, considered one of the main tools to distinguish the inhibition mechanisms involved. Therefore, the inhibition kinetics of the most active *C. bullatus* leaf extract against α-glucosidase and α -amylase were analyzed using the Lineweaver–Burk double reciprocal plots displayed in Figure 2 and Figure 3, and the kinetic parameters (*Vmax* and *Km*) presented in Table 3. The Lineweaver–Burk plots of the investigated extract showed a mixed type of the inhibition for both enzymes studied since increasing the extract concentration resulted in a family of straight lines with different slopes and y-intercepts that intersected in the second quadrant of the graph. Moreover, it may be seen from Figure 2c and Figure 3c that the intersecting lines on the graph converge closer to the X-axis, indicating that although the extract tested is a mixed-type inhibitor, it also has active noncompetitive components. Finally, based on the calculated kinetic parameters, the observed decrease in *Vmax* and increase in *Km* confirm the mixed type of inhibition of both α-glucosidase and α -amylase [26].

Furthermore, in the case of α-amylase, the inhibition type of the tested extract was analogous to the mixed type demonstrated and confirmed in earlier studies for clinically used acarbose [32,33,34]. However, in the α-glucosidase test, the extract with a mixed type of inhibition differed significantly from acarbose, which competitively interacts with this enzyme [35,36,37].

The mixed inhibitors might attach to the enzyme in a different place than the active site, i.e., in the allosteric center and/or indirectly to the enzyme-substrate complex. The result is a change in the conformation of the attachment site that prevents the substrate from binding to the enzyme and thus inhibits the enzymatic reaction [26]. The tested *C. bullatus* extract, as a mixed-type inhibitor, may therefore exhibit two ways of responding: competing with the substrate for binding to the allosteric enzyme center and/or to the existing enzyme-substrate complex [38]. Another advantage of mixed-type inhibitors is their ability to inhibit enzymes independent of substrate concentration. It was reported that with higher carbohydrate intake, the mixed-type inhibitors do not require higher concentration to achieve the same inhibitory effects as those achieved with competitive and noncompetitive inhibitors [38,39].

As previously reported for polyphenols of the same type as found in *C. bullatus* leaf extract, flavonoids and proanthocyanidins show various types of inhibition against digestive enzymes. As was proven for flavonols, rutin showed a mixed type of α-glucosidase inhibition [40]. Similarly, (−)-epicatechin and (+)-catechin were mixed-type inhibitors of α-glucosidase [41,42]. In the case of B2 and B3 procyanidins, their noncompetitive interaction with α-glucosidase was previously demonstrated [43]. Therefore, both the B-type procyanidins and flavonols found as one of the dominant polyphenols of the studied *C. bullatus* extract, through synergistic effects, may be responsible for a mixed type of interaction with α-glucosidase and α-amylase with the suggested noncompetitive inhibition trend.

The development of effective AGE inhibitors is also believed to have therapeutic potential in slowing the development/progression of diabetic complications [44]. In this regard, polyphenol-rich plant extracts are promising because they are among the most effective glycation inhibitors [45,46,47]. In the present study, the *Cotoneaster* leaf and fruit extracts were significant inhibitors of nonenzymatic protein glycation (Table 2). The most effective leaf extracts from *C. bullatus*, *C. zabelii*, and *C. integerrimus* were twice as active as clinically used aminoguanidine. The contribution of leaf and fruit polyphenols to the prevention of AGE formation was confirmed by the statistically significant correlations (Table 4) between the activity results and polyphenolic levels, including polymeric flavan-3-ols (|*r*| = 0.899) and caffeic acid derivatives (|*r*| = 0.790). The synergistic interactions between these polyphenols were probably responsible for the significant inhibitory properties of the tested extracts, even exceeding that of aminoguanidine. Although synthetic aminoguanidine is the prototypical therapeutic agent for preventing AGEs, it has emerging toxic effects; therefore, plant-based remedies with fewer side effects are still required [5]. Previous studies demonstrated the high antiglycation potential of B-type proanthocyanidins, chlorogenic acid isomers, and other caffeic acid derivatives [45,47,48]. These phytochemicals exert their beneficial effects by inhibiting AGE-precursors formation and/or AGE cross linking with collagen in vitro. The proposed mechanism of their protective effects was based on their ability to bind with proteins, block the carbonyl groups of reducing sugars, and break down the AGEs’ structures [6]. The study of the relationship between antiglycation activity and molecular structures suggested that high-molecular-weight proanthocyanidins had stronger antiglycation activity than their corresponding flavan-3-ol monomers and that multiple hydroxyl groups, especially in the position of *meta*- or *ortho*-, are crucial for this activity [49]. This suggestion is in accordance with the present results, which demonstrated dimeric procyanidin B2 as a more effective antiglycation agent than (−)-epicatechin. In a previous study on cinnamic acid derivatives, the observed relatively higher activity for caffeic acid may also be related to the structural differences between caffeic acid and chlorogenic acid, in which the active caffeic acid moiety is ester-bound with quinic acid [45].

## 4. Materials and Methods

### 4.1. Chemical and Reagents

Porcine pancreatic α-amylase, starch soluble from potato, α-glucosidase from *Saccharomyces cerevisiae*, *p*-nitrophenyl-α-D-glucopyranoside (PNPG), *p*-nitrophenol, maltose, acarbose, bovine serum albumin (BSA), fructose and aminoguanidine were purchased from Sigma-Aldrich (Seelze, Germany/St. Louis, MO, USA). The standard of chlorogenic acid hemihydrate (CHA), neochlorogenic acid (NCHA), cryptochlorogenic acid (CCHA), caffeic acid (CA), (–)-epicatechin (ECA), procyanidin C1 (PC1), isoquercitrin (IQ), rutin trihydrate (RT) were purchased from Sigma-Aldrich (St. Louis, MO, USA) or PhytoLab GmbH (Vestenbergsgreuth, Germany). The other standards of caffeoylmalic acid (CAD), procyanidin B2 (PB2), hyperoside (HP), quercitrin (QR), and quercetin 3-*O*-β-D-(2″-*O*-β-D-xylopyranosyl)galactopyranoside (QPH) were previously isolated form the leaves of *C. bullatus* or *C. zabelii* [50]. HPLC-grade solvents such as acetonitrile and phosphoric acid were from Avantor Performance Materials (Gliwice, Poland). All other reagents used in the quantitative study were of analytical grade and of the same origin as described elsewhere for the relevant tests [20,21].

### 4.2. Plant Material and Sample Preparation

The dry leaf and fruit samples of *Cotoneaster bullatus* Bois, *C. zabelii* C.K. Schneid and *C. integerrimus* Medik. were collected and authenticated in the Botanical Garden (51°45′ N 19°24′ E) in Lodz (Poland) or in the Arboretum (51°49′ N 19°53′ E), Forestry Experimental Station of Warsaw University of Life Sciences (SGGW) in Rogow (Poland). The voucher specimens of the leaves (KFG/15/CBLL, KFG/15/CZBL, KFG/15/CINL) and the fruits (KFG/18/CBLF, KFG/18/CZBF, KFG/18/CINF) were deposited in the Herbarium of the Department of Pharmacognosy, Medical University of Lodz (Poland).

The dry samples of the leaves (50 g) and fruits (100 g) were independently defatted by pre-extraction with chloroform in a Soxhlet apparatus (500 mL, 2 h; the chloroform extracts were discarded), and subsequently refluxed with methanol–water, 7:3, *v/v* (3 × 500 mL × 1.5 h). Then, the combined extracts were evaporated in vacuo, separately for each plant material, and the dry, crude extracts were stored at 4 °C for subsequent quantitative and activity analyses.

### 4.3. Quantitative Phytochemical Profiling of Cotoneaster Leaf and Fruit Phenolics

The total phenolic content (TPH) and contents of individual phenolics were quantified using the HPLC-PDA method developed and validated previously [51]. The extract analytes were separated on C18 Ascentis^®^ Express column (2.7 mm, 75 × 4.6 mm i.d.; Supelco, Bellefonte, PA, USA) using a diode array detector operating at 280 nm for flavanols, 325 nm for caffeic acid derivatives, and 350 nm for flavonols. A gradient solvent system consisting of water–orthophosphoric acid, 99.5:0.5, *v/w* (solvent A), acetonitrile (solvent B) and the following elution profile: 0–14 min, 6–30% B (*v/v*); 14–15 min, 30–50% B; 15–17 min, 50%; 17–18 min, 50–6% B; 18–21 min, 6% B (equilibration) was used as mobile phase at a flow rate of 1.4 mL/min and column temperature of 25 °C. The individual analytes were quantified using standards of ECA, PB2, PC1, CHA, CAD, QPH, RT, HP, IQ, and QR. The other polyphenols tentatively identified were quantified as equivalents of PB2 (for proanthocyanidins), CA (for other hydroxycinnamic acid derivatives), HP (for flavonoid monoglycosides), or RT (for flavonoid diglycosides).

The total phenolic content TPC and total proanthocyanidin content TPAC were quantified by the Folin–Ciocalteu and *n*-butanol-HCl methods, respectively, as described previously [51].

### 4.4. α-Glucosidase Inhibitory Assay

The effect of the plant extracts/compounds on yeast α-glucosidase activity was determined according to the method described previously [52] with minor modifications. The *p*-nitrophenyl glucopyranoside (PNPG, 0.2 mg/mL) as reaction substrate was prepared in 0.1 M phosphate buffer (pH = 6.8). The reaction mixture containing α-glucosidase (0.04 mg/mL, 0.43 U/mL, 50 µL) and 150 μL tested extracts/compounds at different concentrations in the 0.1 M phosphate buffer, was incubated for 15 min at 37 °C. Then, to start the enzymatic reaction, 50 μL PNPG (0.2 mg/mL) was added to the mixture and incubated for 15 min at 37 °C. The reaction was stopped by adding 50 μL Na_2_CO_3_ (0.2 M). The extract/compound activity was quantified spectrophotometrically using a SPECTROStar Nano microplate reader (BMG Labtech, Ortenberg, Germany) by measuring the yellow-colored *p*-nitrophenol released from PNPG at 405 nm. The results were expressed as IC_50_ calculated from concentration-inhibition calibration curves (4–5 calibration points). Acarbose (AR), as the commercial inhibitor, was used as the positive control.

### 4.5. α-Amylase Inhibitory Assay

The ability of the plant extracts/compounds to inhibit pancreatic α-amylase was assayed according to the modified procedure of McCue and Shetty [53]. The reaction mixture containing α-amylase (0.2 mg/mL, 2.6 U/mL, 400 µL) and 800 µL tested extracts/compounds at different concentrations in the 0.02 M phosphate buffer (pH = 6.6), was incubated for 15 min at room temperature. Then, the enzymatic reaction was initiated by adding the 400 µL starch solution (1%) to the mixture. After 15 min incubation, the reaction was stopped by adding 300 µL dinitrosalicylic acid reagent (DNS) and 500 µL phosphate buffer (0.02 M, pH = 6.9). The reaction mixture was then heated at 100 °C for 10 min, and after cooling to room temperature, the absorbance was recorded at 540 nm using a UV-1601 Rayleigh spectrophotometer (Beijing, China). The results were expressed as IC_50_ calculated from concentration-inhibition calibration curves (4–5 calibration points). Acarbose (AR), as the commercial inhibitor, was used as the positive control.

### 4.6. Kinetic Parameters of α-Glucosidase and α-Amylase Inhibition

Kinetic parameters of α-glucosidase and α-amylase inhibition were determined for the leaf extract of *C. bullatus*. In the kinetic study, the reaction mixtures were prepared according to the protocols described above (Section 4.4 and Section 4.5). In the inhibition assays, reaction substrates (*S*) were used at different concentrations, including PNPG in the range of 0.28–1.00 mM and soluble starch in the range of 0.26–1.81 mM, for α-glucosidase and α-amylase tests, respectively. The activity of enzymes was evaluated in the absence or presence of different concentrations of the tested extract used as an inhibitor (*I*). The extract concentrations for the α-glucosidase inhibitory kinetics were 5.0, 6.0, and 9.0 µg/mL, whereas 20.0, 40.0, and 60.0 µg/mL were used for α-amylase. The inhibitor concentration ranges were selected based on activity parameters (IC_50_) of the *C. bullatus* leaf extract obtained in the α-glucosidase and α-amylase tests (Table 2). The initial rates of reactions (*V_o_*) were calculated from calibration curves constructed using varying concentrations of *p*-nitrophenol and maltose for the α-glucosidase and α-amylase assay, respectively.

In order to assess the interaction type between enzymes (α-glucosidase and α-amylase) and their inhibitor (the *C. bullatus* leaf extract), a nonlinear Michaelis–Menten regression plot (*V* versus *S*) and a corresponding Lineweaver–Burk double reciprocal plot (*1/V* versus *1/S*) were constructed for each concentration of inhibitor (*I*) and enzyme reaction substrate (*S*). Based on the Lineweaver–Burk graph, two important terms in enzyme kinetics could be determined: *Km* (Michaelis constant) and *Vmax* (Maximum velocity). The *y*-intercept of such a graph corresponds to the inverse of *Vmax*, while the *x*-axis intersection represents −1/*Km*. *Vmax* and *Km* could therefore be determined experimentally or calculated from the Lineweaver-Burk equation [26]:

(1) $$\frac{1}{V}=\frac{Km}{Vmax}\;\times \;\frac{1}{S}+\frac{1}{Vmax}$$

where *Km* is the Michaelis constant; *Vmax* is the maximum velocity; *V* is the reaction velocity, and *S* is the substrate of the enzymatic reaction.

The study of the inhibition type of the tested extract (competitive, noncompetitive, or mixed type) was performed by the comparison of *Km* and *Vmax* and their ratio (*Km*/V*max*) in the presence and absence of the inhibitor (*I*). The type of inhibition was also assessed based on the graphical view of the Lineweaver–Burk plot (1/*V* versus 1/*S*) [26].

### 4.7. Protein Glycation Inhibitory Assay

The ability of the plant extracts/compounds to inhibit fructose-induced protein glycation was determined according to a method previously published [54] with minor modifications. A solution of bovine serum albumin (BSA, 10 mg/mL) and fructose (90 mg/mL) was prepared in phosphate buffer (0.1 mM, pH 7.4) containing 0.01% of NaN_3_ as antimicrobial agent. Briefly, fructose solution (600 µL) was mixed with BSA (600 µL) and 600 µL tested extracts/compounds at different concentrations and then the reaction mixture was incubated in the capped amber vials in the dark at 37 °C for six days. After incubation, all samples were transferred to 96-well black microplates (Greiner, Germany) to measure glycated BSA formation using a multilabel counter Victor 1420 (Perkin Elmer Life and Analytical Sciences, Shelton, CT, USA) at excitation and emission wavelengths of 355 nm and 420 nm, respectively. The results were expressed as IC_50_, calculated from concentration-inhibition curves (4–5 calibration points). Aminoguanidine (AG), as the commercial inhibitor, was used as the positive control.

### 4.8. Statistical Analysis

All samples were assayed in triplicate, and results are given as the mean ± SD (standard deviation) using Microsoft Excel XP. The statistical significance of differences between the means was calculated using one-way variance analysis (ANOVA) followed by a post hoc Tukey test. All calculations were performed using Statistica 13.1Pl software (StatSoft, Krakow, Polska). Statistically significant differences were set at *p* < 0.05.

## 5. Conclusions

The present study is the first evaluation of *C. bullatus*, *C, zabelii*, and *C. integerrimus* leaf and fruit extracts as natural products with potential value for their use as functional food ingredients in the prevention of diabetes mellitus. The current results suggest that one of the mechanisms by which the methanol–water (7:3, *v/v*) *Cotoneaster* extracts might reveal hypoglycemic potential is their ability to inhibit the digestive enzymes and the production of AGE products. In addition, based on the correlation studies, proanthocyanidins were indicated as crucial determinants of the tested activity. However, considering the significant contribution of these compounds to the extracts’ composition, further detailed studies are required to explore their molecular structures and directly confirm their postulated activity, including possible in vivo effects as well as bioavailability and safety.

## Figures and Tables

**Table 1:** The quantitative profile of polyphenols in the methanol–water (7:3, *v*/*v*) extracts of *C. bullatus*, *C. zabelii*, and *C. integerrimus* fruits and leaves.

	MBL	MZL	MIL	MBF	MZF	MIF
Individual analyte			mg/g dw			
NCHA	3.71 ± 0.10 ^C^	1.99 ± 0.01 ^A^	2.66 ± 0.06 ^B^	0.79 ± 0.00 ^E^	0.09 ± 0.00 ^A^	0.70 ± 0.00 ^C^
CHA	17.07 ± 0.88 ^H^	9.24 ± 0.11 ^F^	61.53 ± 0.80 ^F^	2.36 ± 0.00 ^K^	2.17 ± 0.07 ^C^	1.52 ± 0.03 ^E^
CCHA	3.64 ± 0.15 ^C^	2.70 ± 0.03 ^B^	2.78 ± 0.01 ^B^	0.42 ± 0.01 ^C^	0.26 ± 0.02 ^A,B^	0.29 ± 0.01 ^A^
PB2	18.45 ± 1.00 ^I^	12.09 ± 0.15 ^G^	n.d.	1.42 ± 0.02 ^I^	4.93 ± 0.08 ^E^	2.86 ± 0.16 ^F^
ECA	12.62 ± 0.65 ^F^	8.98 ± 0.10 ^E^	n.d.	1.28 ± 0.01 ^H^	6.99 ± 0.12 ^F^	2.94 ± 0.12 ^F^
PC1	15.04 ± 0.76 ^G^	8.95 ± 0.06 ^E^	n.d.	1.10 ± 0.03 ^F^	4.22 ± 0.20 ^D^	3.13 ± 0.04 ^G^
CAD	n.d.	32.05 ± 0.25 ^H^	n.d.	n.d.	n.d.	n.d.
QPH	7.75 ± 0.32 ^E^	n.d.	n.d.	1.59 ± 0.02 ^J^	n.d.	n.d.
RT	0.27 ± 0.01 ^A^	3.35 ± 0.01 ^C^	4.33 ± 0.07 ^C^	0.18 ± 0.00 ^A^	0.29 ± 0.01 ^B^	0.98 ± 0.02 ^D^
HP	5.86 ± 0.15 ^D^	1.73 ± 0.03 ^A^	39.17 ± 0.43 ^E^	1.16 ± 0.00 ^G^	0.33 ± 0.01 ^B^	1.48 ± 0.02 ^E^
IQ	0.83 ± 0.05 ^B^	2.38 ± 0.03 ^B^	20.38 ± 0.22 ^D^	0.35 ± 0.00 ^B^	0.27 ± 0.01 ^A,B^	0.47 ± 0.02 ^B^
QR	3.42 ± 0.18 ^C^	4.36 ± 0.05 ^D^	1.72 ± 0.03 ^A^	0.62 ± 0.02 ^D^	0.21 ± 0.01 ^A,B^	0.22 ± 0.00 ^A^
Phenolic fraction			mg/g dw			
TPC (GAE)	316.27 ± 3.94	337.63 ± 2.33	200.30 ± 2.90	62.13 ± 3.15	77.86 ± 2.07	81.26 ± 0.77
TPA (CYE)	135.32 ± 1.88	147.91 ± 0.50	86.64 ± 2.42	20.42 ± 1.16	34.11 ± 0.44	35.68 ± 0.87
TPH	100.42 ± 4.92	103.10 ± 1.00	134.89 ± 1.65	12.18 ± 0.05	22.27 ± 0.11	15.47 ± 0.42
TCA	24.42 ± 1.02	43.97 ± 0.44	66.97 ± 0.85	3.57 ± 0.01	2.52 ± 0.05	2.52 ± 0.03
TFL	19.62 ± 0.85	14.94 ± 0.10	67.91 ± 0.80	4.01 ± 0.01	1.31 ± 0.03	3.76 ± 0.07
TLPA	56.38 ± 2.86	44.19 ± 0.45	n.d.	3.79 ± 0.04	17.50 ± 0.24	8.93 ± 0.32

Data expressed as mean ± SD (*n* = 3); for each analyte; different superscript letters indicate significant differences (*p* < 0.05); the methanol–water (7:3, *v/v*) extracts of the leaves and fruits of *C. bullatus* (MBL, MBF), *C. zabelii* (MZL, MZF), and *C. integerrimus* (MIL, MIF); TPC, total phenolic content in gallic acid equivalent (GAE) detemined by Folin–Ciocalteu assay; TPA, total proanthocyanidin content in cyanidin chloride equivalent (CYE) determined by *n*-butanol/HCl assay; TPH, total phenolic content determined by RP-HPLC-PDA as a sum of individual compounds; TCA, total caffeic acid derivatives content; TFL, total flavonoid content; TLPA, total content of low-molecular flavanols and proanthocyanidins; NCHA, 3-*O*-caffeoylquinic acid; CCHA, 4-*O*-caffeoylquinic acid; CHA, 5-*O*-caffeoylquinic acid (chlorogenic acid); CAD, caffeoylmalic acid; ECA, (–)-epicatechin; PB2, PC1, procyanidins B2, C1, respectively; QPH, quercetin 3-(2″-xylosyl)galactoside; RT, rutin; HP, hyperoside; IQ, isoquercitrin; QR, quercitrin; additional abbreviations: n.d., not detected.

**Table 2:** Inhibitory effects (IC_50_) of *Cotoneaster* leaf and fruit extracts and individual phenolics on α-glucosidase, α-amylase, and protein glycation.

Analyte	α-Glucosidase	α-Amylase	Protein Glycation
Phenolic Extracts			
	µg/mL	µg/mL	µg/mL
MBL	8.56 ± 0.32 ^A^	41.82 ± 1.78 ^A^	32.62 ± 3.71 ^C^
MZL	9.53 ± 0.33 ^A^	33.03 ± 1.63 ^A^	36.53 ± 3.64 ^C^
MIL	53.92 ± 3.48 ^B^	1511.41 ± 50.23 ^E^	36.28 ± 2.35 ^C^
MBF	80.12 ± 3.91 ^C^	941.13 ± 49.98 ^C^	166.62 ± 7.71 ^G^
MZF	57.73 ± 2.86 ^B^	1082.52 ± 43.33 ^D^	118.94 ± 8.82 ^F^
MIF	48.89 ± 4.81 ^B^	976.20 ± 6.65 ^C^	106.36 ± 6.47 ^E^
Pure Compounds/Standards			
	µg/mL	µg/mL	µg/mL
PB2	416.37 ± 11.23 ^H^	>200	2.20 ± 0.01 ^A^
ECA	231.66 ± 8.09 ^E^	>200	8.79 ± 0.13 ^A, B^
CAD	43.70 ± 2.18 ^B^	>400	17.26 ± 1.09 ^B^
QPH	311.80 ± 1.97 ^G^	>400	15.78 ± 1.14 ^A, B^
HP	283.48 ± 3.47 ^F^	>400	14.34 ± 1.15 ^A, B^
AR	169.52 ± 9.45 ^D^	5.78 ± 0.27 ^A^	n.d.
AG	n.d.	n.d.	71.09 ± 4.20 ^D^

Codification of the phenolic compounds and extracts in Table 1; positive control: AR—acarbose, AG—aminoguanidine; results are expressed as means ± SD (*n* = 3); for each test, different superscript letters indicate significant differences (*p* < 0.05); additional abbreviations: n.d., not detected.

**Table 3:** Kinetic parameters of α-amylase and α-glucosidase in the presence of *C. bullatus* leaf extract and acarbose.

Inhibitor	Concentration of Inhibitor (µg/mL)	*V_max_* (mM/min)		*K_m_* (mM)		V*_max_*/*K_m_*	Type Inhibition
α-glucosidase							
MBL	no inhibitor	***V_maxo_***	0.024 ± 0.001 ^D^	***K_mo_***	0.454 ± 0.020 ^A^	0.048 ^D^	Mixed
	5.0	***V_max1_***	0.020 ± 0.00 1^C^	***K_m1_***	0.533 ± 0.010 ^A,B^	0.038 ^C^	
	6.0	***V_max2_***	0.016 ± 0.001 ^B^	***K_m2_***	0.592 ± 0.030 ^C^	0.027 ^B^	
	9.0	***V_max3_***	0.008 ± 0.001 ^A^	***K_m3_***	0.780 ± 0.040 ^D^	0.012 ^A^	
AR	no inhibitor	***V_maxo_***	0.029 ± 0.001^A^	***K_mo_***	0.280 ± 0.015 ^A^	0.136 ^C^	Competitive
	110.0	***V_max1_***	0.028 ± 0.001^A^	***K_m1_***	0.867 ± 0.010 ^B^	0.032 ^B^	
	160.0	***V_max2_***	0.027 ± 0.001^A^	***K_m2_***	1.086 ± 0.010 ^C^	0.025 ^A,B^	
	215.0	***V_max3_***	0.029 ± 0.001^A^	***K_m3_***	1.370 ± 0.020 ^D^	0.021 ^A^	
α-amylase							
MBL	no inhibitor	***V_maxo_***	0.465 ± 0.009 ^C^	***K_mo_***	0.860 ± 0.050 ^A^	0.541 ^D^	Mixed
	20.0	***V_max1_***	0.333 ± 0.011 ^B^	***K_m1_***	1.249 ± 0.045 ^B^	0.267 ^C^	
	40.0	***V_max2_***	0.276 ± 0.015 ^A,B^	***K_m2_***	1.360 ± 0.030 ^C^	0.203 ^B^	
	60.0	***V_max3_***	0.226 ± 0.001 ^A^	***K_m3_***	1.515 ± 0.025 ^D^	0.149 ^A^	
AR	no inhibitor	***V_maxo_***	0.382 ± 0.020 ^C^	***K_mo_***	0.873 ± 0.009 ^A^	0.438 ^D^	Mixed
	3.0	***V_max1_***	0.321 ± 0.009 ^C^	***K_m1_***	0.906 ± 0.009 ^B^	0.354 ^C^	
	8.0	***V_max2_***	0.241 ± 0.015 ^B^	***K_m2_***	0.986 ± 0.009 ^C^	0.326 ^B^	
	10.0	***V_max3_***	0.189 ± 0.009 ^A^	***K_m3_***	1.274 ± 0.009 ^D^	0.148 ^A^	

MBL, methanol–water (7:3, *v/v*) extract of *C. bullatus* leaves; AR, acarbose; *V_max_*_,_ maximum velocity of enzymatic activity; *K*_m_, Michaelis constant; the kinetic parameters (*V_max_*, *K_m_*) at control, and at three different concentrations of inhibitors (MBL, AR) were calculated based on Lineweaver–Burk plot; data expressed as mean ± SD (*n* = 3); for each inhibitor, different superscript letters indicate significant differences (*p* < 0.05).

**Table 4:** Correlation coefficients (*r*) and probability (*p*) of the linear relationships between phenolic contents of *Cotoneaster* leaf and fruit extracts and their activity parameters—influence on α-glucosidase, α-amylase inhibition, and AGE formation.

*r* (*p*) for:	TPC (mg GAE/g)	TPA (mg CYE/g)	TPH (mg/g)	TCA (mg/g)	TFL (mg/g)	TLPA (mg/g)
α-glucosidase (µg/mL)	**−0.9119** (0.011) *	**−0.9216** (0.009) *	−0.5819 (0.226)	−0.3844 (0.452)	−0.0648 (0.903)	**−0.8957** (0.016) *
α-amylase (µg/mL)	**−0.7301** **(0.039)** *	**−0.7205** (0.048) *	−0.1924 (0.715)	0.0055 (0.993)	0.3719 (0.468)	**−0.9274** (0.008) *
protein glycation (µg/mL)	**−0.8854** (0.019) *	**−0.8988** (0.015) *	**−0.9121** (0.011) *	**−0.7899** (0.042) *	−0.6140 (0.195)	−0.5612 (0.240)

Activity and concentration parameters according to Table 1 and Table 2; asterisks mean statistical significance of the estimated linear relationships (* *p* < 0.05); all positive correlations are printed in bold.

## Data Availability

Data is contained within the article.

## References

[1] Khan M.A.B., Hashim M.J., King J.K., Govender R.D., Mustafa H., Al Kaabi J. (2020). Epidemiology of Type 2 diabetes-global burden of disease and forecasted trends. J. Epidemiol. Glob. Health.

[2] Twarda-Clapa A., Olczak A., Białkowska A.M., Koziołkiewicz M. (2022). Advanced glycation end-products (AGEs): Formation, chemistry, classification, receptors, and diseases related to AGEs. Cells.

[3] Nowotny K., Jung T., Höhn A., Weber D., Grune T. (2015). Advanced glycation end products and oxidative stress in type 2 diabetes mellitus. Biomolecules.

[4] Papoutsis K., Zhang J., Bowyer M.C., Brunton N., Gibney E.R., Lyng J. (2021). Fruit, vegetables, and mushrooms for the preparation of extracts with α-amylase and α-glucosidase inhibition properties: A review. Food Chem..

[5] Goh S.Y., Cooper M.E. (2008). The role of advanced glycation end products in progression and complications of diabetes. J. Clin. Endocrinol. Metab..

[6] Song Q., Liu J., Dong L., Wang X., Zhang X. (2021). Novel advances in inhibiting advanced glycation end product formation using natural compounds. Biomed. Pharmacother..

[7] Lee J., Noh S., Lim S., Kim B. (2021). Plant extracts for type 2 diabetes: From traditional medicine to modern drug discovery. Antioxidants.

[8] Willcox M.L., Elugbaju C., Al-Anbaki M., Lown M., Graz B. (2021). Effectiveness of medicinal plants for glycaemic control in type 2 diabetes: An overview of meta-analyses of clinical trials. Front. Pharmacol..

[9] Dirir A.M., Daou M., Yousef A.F., Yousef L.F. (2022). A review of alpha-glucosidase inhibitors from plants as potential candidates for the treatment of type-2 diabetes. Phytochem. Rev..

[10] Alam F., Shafique Z., Amjad S.T., Bin Asad M.H.H. (2019). Enzymes inhibitors from natural sources with antidiabetic activity: A review. Phytother. Res..

[11] Ahangarpour A., Sayahi M., Sayahi M. (2019). The antidiabetic and antioxidant properties of some phenolic phytochemicals: A review study. Diabetes Metab. Syndr..

[12] Sun L., Miao M. (2020). Dietary polyphenols modulate starch digestion and glycemic level: A review. Crit. Rev. Food Sci. Nutr..

[13] Da Porto A., Cavarape A., Colussi G., Casarsa V., Catena C., Sechi L.A. (2021). Polyphenols rich diets and risk of type 2 diabetes. Nutrients.

[14] Kicel A. (2020). An Overview of the Genus *Cotoneaster* (Rosaceae): Phytochemistry, Biological Activity, and Toxicology. Antioxidants.

[15] Holzer V.M.D., Lower-Nedza A.D., Nandintsetseg M., Batkhuu J., Brantner A.H. (2013). Antioxidant constituents of *Cotoneaster* melanocarpus Lodd. Antioxidants.

[16] Les F., López V., Caprioli G., Iannarelli R., Fiorini D., Innocenti M., Bellumori M., Maggi F. (2017). Chemical constituents, radical scavenging activity and enzyme inhibitory capacity of fruits from *Cotoneaster pannosus* Franch. Food Funct..

[17] Zengin G., Uysal A., Gunes E., Aktumsek A. (2014). Survey of phytochemical composition and biological effects of three extracts from a wild plant (*Cotoneaster nummularia* Fisch. et Mey.): A potential source for functional food ingredients and drug formulations. PLoS ONE.

[18] Uysal A., Zengin G., Mollica A., Gunes E., Locatelli M., Yilmaz T., Aktumsek A. (2016). Chemical and biological insights on *Cotoneaster integerrimus*: A new (−)-epicatechin source for food and medicinal applications. Phytomedicine.

[19] Mohamed S.A., Sokkar N.M., El-Gindi O., Ali Z.Y., Alfishawy I.A. (2012). Phytoconstituents investigation, anti-diabetic and anti-dyslipidemic activities of *Cotoneaster horizontalis* Decne cultivated in Egypt. Life Sci. J..

[20] Kicel A., Kolodziejczyk-Czepas J., Owczarek A., Marchelak A., Sopinska M., Ciszewski P., Nowak P., Olszewska M.A. (2018). Polyphenol-rich extracts from *Cotoneaster* leaves inhibit pro-inflammatory enzymes and protect human plasma components against oxidative stress in vitro. Molecules.

[21] Kicel A., Kolodziejczyk-Czepas J., Owczarek A., Rutkowska M., Wajs-Bonikowska A., Granica S., Nowak P., Olszewska M.A. (2018). Multifunctional phytocompounds in *Cotoneaster* fruits: Phytochemical profiling, cellular safety, anti-inflammatory and antioxidant effects in chemical and human plasma models in vitro. Oxid. Med. Cell. Longev..

[22] Kicel A., Owczarek A., Gralak P., Ciszewski P., Olszewska M.A. (2019). Polyphenolic profile, antioxidant activity, and pro-inflammatory enzymes inhibition of leaves, flowers, bark and fruits of *Cotoneaster integerrimus*: A comparative study. Phytochem. Lett..

[23] Wu J., Xia S., Kalionis B., Wan W., Sun T. (2014). The role of oxidative stress and inflammation in CVD aging. Biomed. Res. Int..

[24] Zhang Y.J., Gan R.Y., Li S., Zhou Y., Li A.N., Xu D.P., Li H.B. (2015). Antioxidant phytochemicals for the prevention and treatment of chronic diseases. Molecules.

[25] Rodriguez J.-M.G., Hux N.P., Philips S.J., Towns M.H. (2019). Michaelis–Menten graphs, Lineweaver–Burk plots, and reaction schemes: Investigating introductory biochemistry students’ conceptions of representations in enzyme kinetics. J. Chem. Educ..

[26] Aledo J.C. (2021). Enzyme kinetic parameters estimation: A tricky task?. Biochem. Mol. Biol. Educ..

[27] Adisakwattana S., Jiphimai P., Prutanopajai P., Chanathong B., Sapwarobol S., Ariyapitipan T. (2010). Evaluation of alpha-glucosidase, alpha-amylase and protein glycation inhibitory activities of edible plants. Int. J. Food Sci. Nutr..

[28] Elya B., Basah K., Mun’im A., Yuliastuti W., Bangun A., Septiana E.K. (2012). Screening of α-glucosidase inhibitory activity from some plants of Apocynaceae, Clusiaceae, Euphorbiaceae, and Rubiaceae. J. Biomed. Biotechnol..

[29] Smeriglio A., Barreca D., Bellocco E., Trombetta D. (2017). Proanthocyanidins and hydrolysable tannins: Occurrence, dietary intake and pharmacological effects. Br. J. Pharmacol..

[30] Boath A.S., Stewart D., McDougall G.J. (2012). Berry components inhibit α-glucosidase in vitro: Synergies between acarbose and polyphenols from black currant and rowanberry. Food Chem..

[31] Bräunlich M., Slimestad R., Wangensteen H., Brede C., Malterud K.E., Barsett H. (2013). Extracts, anthocyanins and procyanidins from *Aronia melanocarpa* as radical scavengers and enzyme inhibitors. Nutrients.

[32] Al Kazaz M., Desseaux V., Marchis-Mouren G., Prodanov E., Santimone M. (1998). The mechanism of porcine pancreatic alpha-amylase. Inhibition of maltopentaose hydrolysis by acarbose, maltose and maltotriose. Eur. J. Biochem..

[33] Yoon S.H., Robyt J.F. (2003). Study of the inhibition of four alpha amylases by acarbose and its 4IV-α-maltohexaosyl and 4IV-α-maltododecaosyl analogues. Carbohydr. Res..

[34] Ferey-Roux G., Perrier J., Forest E., Marchis-Mouren G., Puigserver A., Santimone M. (1998). The human pancreatic α-amylase isoforms: Isolation, structural studies and kinetics of inhibition by acarbose. Biochim. Biophys. Acta.

[35] Bischoff H. (1995). The mechanism of alpha-glucosidase inhibition in the management of diabetes. Clin. Investig. Med..

[36] Yan J., Zhang G., Pan J., Wang Y. (2014). α-Glucosidase inhibition by luteolin: Kinetics, interaction and molecular docking. Int. J. Biol. Macromol..

[37] Chougale A.D., Ghadyale V.A., Panaskar S.N., Arvindekar A.U. (2009). Alpha glucosidase inhibition by stem extract of *Tinospora cordifolia*. J. Enzym. Inhib. Med. Chem..

[38] Zhang H., Wang G., Dong J. (2015). Inhibitory properties of aqueous ethanol extracts of propolis on alpha-glucosidase. Evid. Based Complement. Alternat. Med..

[39] Ghadyale V., Takalikar S., Haldavnekar V., Arvindekar A. (2012). Effective Control of Postprandial Glucose Level through Inhibition of Intestinal Alpha Glucosidase by Cymbopogon martinii (Roxb.). Evid. Based Complement. Alternat. Med..

[40] Li Y.Q., Zhou F.C., Gao F., Bian J.S., Shan F. (2009). Comparative evaluation of quercetin, isoquercetin and rutin as inhibitors of alpha-glucosidase. J. Agric. Food Chem..

[41] Wu X., Hu M., Hu X., Ding H., Gong D., Zhang G. (2019). Inhibitory mechanism of epicatechin gallate on α-amylase and α-glucosidase and its combinational effect with acarbose or epigallocatechin gallate. J. Mol. Liq..

[42] Nirmal N.P., Benjakul S. (2012). Inhibition kinetics of catechin and ferulic acid on polyphenoloxidase from cephalothorax of Pacific white shrimp (*Litopenaeus vannamei*). Food Chem..

[43] Sylvain G., Patrice P., Jean-Marc B., Véronique C. (1996). Inhibition of β-glucosidase (*Amygdalae dulces*) by (+)-catechin oxidation products and procyanidin dimers. Biosci. Biotechnol. Biochem..

[44] Sadowska-Bartosz I., Bartosz G. (2015). Prevention of protein glycation by natural compounds. Molecules.

[45] Kim J., Jeong I.H., Kim C.S., Lee Y.M., Kim J.M., Kim J.S. (2011). Chlorogenic acid inhibits the formation of advanced glycation end products and associated protein cross-linking. Arch. Pharm. Res..

[46] Jang D.S., Yoo N.H., Kim N.H., Lee Y.M., Kim C.S., Kim J., Kim J.S. (2010). 3,5-Di-O-caffeoyl-epi-quinic acid from the leaves and stems of *Erigeron annuus* inhibits protein glycation, aldose reductase, and cataractogenesis. Biol. Pharm. Bull..

[47] Gugliucci A., Bastos D.H.M., Schulze J., Souza M.F.F. (2009). Caffeic and chlorogenic acids in *Ilex paraguariensis* extracts are the main inhibitors of AGE generation by methylglyoxal in model proteins. Fitoterapia.

[48] Bains Y., Gugliucci A. (2017). Ilex paraguariensis and its main component chlorogenic acid inhibit fructose formation of advanced glycation endproducts with amino acids at conditions compatible with those in the digestive system. Fitoterapia.

[49] Xie Y., Chen X. (2013). Structures required of polyphenols for inhibiting advanced glycation end products formation. Curr. Drug Metab..

[50] Kicel A., Owczarek A., Kapusta P., Kolodziejczyk-Czepas J., Olszewska M.A. (2020). Contribution of Individual Polyphenols to Antioxidant Activity of *Cotoneaster bullatus* and *Cotoneaster zabelii* Leaves—Structural Relationships, Synergy Effects and Application for Quality Control. Antioxidants.

[51] Olszewska M.A., Michel P. (2009). Antioxidant activity of inflorescences, leaves and fruits of three Sorbus species in relation to their polyphenolic composition. Nat. Prod. Res..

[52] Kim Y.-M., Jeong Y.-K., Wang M.-H., Lee W.-Y., Rhee H.-I. (2005). Inhibitory effect of pine extract on α-glucosidase activity and postprandial hyperglycemia. Nutrition.

[53] McCue P.P., Shetty K. (2004). Inhibitory effects of rosmarinic acid extracts on porcine pancreatic amylase in vitro. Asia Pac. J. Clin. Nutr..

[54] Rutkowska M., Kolodziejczyk-Czepas J., Owczarek A., Zakrzewska A., Magiera A., Olszewska M.A. (2021). Novel insight into biological activity and phytochemical composition of Sorbus aucuparia L. fruits: Fractionated extracts as inhibitors of protein glycation and oxidative/nitrative damage of human plasma components. Food Res. Int..

